# The effect of tobacco and alcohol and their reduction/cessation on mortality in oral cancer patients: short communication

**DOI:** 10.1186/1758-3284-4-6

**Published:** 2012-03-12

**Authors:** Waseem Jerjes, Tahwinder Upile, Hani Radhi, Aviva Petrie, Jesuloba Abiola, Aidan Adams, Panagiotis Kafas, Jacqueline Callear, Ramin Carbiner, Kartic Rajaram, Colin Hopper

**Affiliations:** 1 UCL Department of Surgery, University College London Medical School, London, UK; 2 Oral and Maxillofacial Surgery Unit, AL-Mustansirya University, Baghdad, Iraq; 3 Leeds Institute of Molecular Medicine, School of Medicine, University of Leeds, Leeds, UK; 4 Department of Otolaryngology/Head and Neck Surgery, Chase Farm & Barnet NHS Trust, Enfield, UK; 5 Head & Neck Unit, University College London Hospital, London, UK; 6 Biostatistics Unit, UCL Eastman Dental Institute, London, UK; 7 Department of Oral Surgery and Radiology, School of Dentistry, Aristotle University, Thessalonica, Greece

## Abstract

**Background:**

The use of tobacco is known to increase the incidence of developing oral cancer by 6 times, while the additive effect of drinking alcohol further increases the risk leading to higher rate of morbidity and mortality. In this short communication, we prospectively assessed the effect of tobacco smoking and alcohol drinking in oral cancer patients on the overall mortality from the disease, as well as the effect of smoking and drinking reduction/cessation at time of diagnosis on mortality in the same group.

**Materials and methods:**

A cohort, involved 67 male patients who were diagnosed with oral squamous cell carcinoma, was included in this study. The smoking and drinking habits of this group were recorded, in addition to reduction/cessation after diagnosis with the disease. Comparisons were made to disease mortality at 3 and 5 years.

**Results:**

Follow-up resulted in a 3-year survival of 46.8% and a 5-year survival of 40.4%. Reduction of tobacco smoking and smoking cessation led to a significant reduction in mortality at 3 (P < 0.001) and 5 (P < 0.001) years. Reduction in drinking alcohol and drinking cessation led to a significant reduction in mortality at 3 (P < 0.001) and 5 (P < 0.001) years.

**Conclusion:**

Chronic smoking and drinking does have an adverse effect on patients with oral cancer leading to increased mortality from cancer-related causes. Reduction/cessation of these habits tends to significantly reduce mortality in this group of patients. Smoking and drinking cessation counseling should be provided to all newly diagnosed oral cancer patients.

## Introduction

Cancers of the oral cavity and the oro-pharyngeal region, a subtype of head and neck, continue to be the 6^th ^most common cancer in the world. They may arise as a primary disease or as a secondary disease by extension from regional or distant areas. The most affected oral sites include the oral tongue, floor of mouth and buccal mucosa [1-5].

This disease is known to affect more males than females, and most commonly reported in the elderly population. The effect of human papilloma virus (HPV) has led to increase in the reports of oral cancer in younger population. There are a variety of known histological subtypes, but squamous cell carcinomas (SCC) arises in up to 90% of the cases [6-8].

The exact aetiology behind oral cancer is not fully understood, but mutations at the DNA level usually lead to activation of certain oncogenes causing abnormal growth. The most predominant risk factors include tobacco use and alcohol consumption. These risk factors may cause or contribute to the formation of oral leukoplakia, erthyroplakia, speckled leukoplakia and other abnormal premalignant oral disorders including submucous fibrosis. Nowadays, the current trends in the spread of HPV16 have lead in more oral cancer being diagnosed in non-smokers and non-drinkers [9-11].

The literature is full of case-control and cohort analytic studies establishing a fundamental relationship between patients using tobacco and alcohol and developing oral cancer. Smoking tobacco is known to increase the incidence of developing oral cancer by 6 times, while the additive effect of drinking alcohol further increases the risk leading to higher rate of morbidity and mortality [12-23].

Chronic smokers and drinkers are known to succumb from this disease much faster than non-smokers, non-drinkers oral cancer patients. Also, chewing betel, paan and Areca is known to be risk factor as well. Smokeless tobacco contains nicotine and nitrosamines and is usually marketed for oral or nasal use; several studies have provided evidence linking it to oral cancer [12-23].

In this short communication, a small cohort of patients with oral squamous cell carcinoma (SCC) that underwent surgical resection for their pathologies were followed up for a minimum period of 5 years. The aim of this study was to prospectively assess the effect tobacco and alcohol consumption on mortality from this disease; also the effect of smoking and alcohol reduction/cessation at time of diagnosis on survival.

## Materials and methods

In this prospective study, identical treatment protocols were used to treat 67 patients with oral SCC referred to the Department of Oral and Maxillofacial Surgery, University College Hospital, London between 1998 and 2003.

All applications were accompanied by multidisciplinary team recommendation, ethical approval, and informed patient consent. The patients' data were entered onto proformas, which were validated and checked by interval sampling. The patients' data included a range of clinical and operative variables. Data collection also included smoking and drinking status, smoking and drinking reduction/cessation at time of diagnosis, comorbidities, recurrence, cause of death, date of death, and last clinic review. The wording "chronic" was used for smokers and drinkers with an ongoing habit for > 20 years.

Demographics of the patients included in the study are highlighted in Table 1. The patient population comprised 67 males. Their mean age at the first diagnosis of Oral SCC was 62.2 years (SD 15.6, min. 25, max. 96 years). Most of the patients were Caucasians (82.1%); other prominent racial groups included Indians (10.4%) and Middle-Easterns (4.5%).

**Table 1:** Demographics of the patients included in the study

	No. of patients (%)		No. of patients (%)		No. of patients (%)
Age at 1st presentation		Secondary site (Cont.)		Tumour clearance	
Mean	62.2	Maxillary tuberosity	1 (1.5)	Positive margins	28 (41.8)
Std. Deviation	15.6	Retromolar area	1 (1.5)	Negative margins	39 (58.2)
Minimum	24.0	Hard palate	1 (1.5)		
Maximum	96.0	Buccal mucosa	5 (7.5)	**Depth of invasion (mm)**	
		Tonsil	2 (3.0)	Median	10.0
**ASA**				Minimum	1.2
I	24 (35.8)	**Smoking status**		Maximum	35.0
II	31 (46.3)	Non-smoker	12 (17.9)		
III	12 (17.9)	Ex-smoker	6 (9.0)	**Dysplasia at margin**	19 (28.4)
		Chronic Smoker (< 5 cig/day)	3 (5.5)		
**Ethnicity**		Chronic Smoker 9 cig/day)	(5- 6 (10.9)	**Presence of severe dysplasia**	33 (49.3)
Caucasian	55 (82.1)	Chronic Smoker (10-14 cig/day)	1 (1.8)		
Indian	7 (10.4)	Chronic Smoker (15-19 cig/day)	1 (1.8)	**Non-cohesive Invasive front**	34 (50.7)
Middleastern	3 (4.5)	Chronic Smoker (≥ 20 cig/day)	37 (67.3)		
African	2 (3.0)			**Vascular invasion**	3 (4.5)
		**Advice on smoking cessation**			
**Clinical presentation**		Reduction of smoking	12 (21.8)	**Nerve invasion**	1 (1.5)
Ulcer	50 (74.6)	Cessation of Smoking	13 (23.6)		
Macule	2 (3.0)			**Bone/cartilage invasion**	0 (0.0)
Papule	14 (20.9)	**Betel nut chewing**	6 (9.0)		
Nodule	1 (1.5)			**Days in ICU**	
		**Drinking status**		Median	1
**Primary site**		Non-drinker	20 (29.9)	Minimum	0
Lateral tongue	25 (37.3)	Ex-drinker	1 (1.5)	Maximum	11
FOM	14 (20.9)	Chronic drinker (< 10 u/week)	2 (4.3)		
Lower alveolus	8 (11.9)	Chronic drinker (10-20 u/week)	9 (19.1)	**Days in Hospital**	
Retromolar area	4 (6.0)	Chronic drinker (> 20 u/week)	35 (74.5)	Median	12
Upper alveolus	4 (6.0)			Minimum	1
Dorsal tongue	1 (1.5)	**Advice on drinking cessation**		Maximum	90
Buccal mucosa	5 (7.5)	Alcohol reduction	15 (31.9)		
Ventral tongue	1 (1.5)	Alcohol cessation	9 (19.1)	**Post-surgical radiotherapy**	40 (59.7)
Lower lip	2 (3.0)				
Soft palate	1 (1.5)	**Tumour staging**		**Recurrence**	26 (38.8)
Hard palate	1 (1.5)	I	13 (19.4)		
Tonsil	1 (1.5)	II	10 (14.9)	**Survived 3 years**	22 (46.8)
		III	3 (4.5)		
**Secondary site**		IV	41 (61.2)	**Survived 5 years**	19 (40.4)
No site	44 (65.7)				
Lower alveolus	5 (7.5)	**Differentiation**			
Ventral tongue	2 (3.0)	Well	13 (19.4)		
Soft palate	1 (1.5)	Well-moderate	4 (6.0)		
FOM	5 (7.5)	Moderate	40 (59.7)		
		Moderate-poor	5 (7.5)		
		Poor	5 (7.5)		

Clinical presentation was mostly an ulcer (74.6%) or a papule (20.9%). Primary sites were mainly identified in the tongue (40.3%), floor of mouth (FOM, 20.9%), lower alveolus (11.9%) and buccal mucosa (7.5%).

Tobacco smoking status was categorised into non-smokers (17.9%), ex-smokers (9.0%), and 5 categories of chronic smokers (i) < 5 cig/day (5.5%), (ii) 5-9 cig/day (10.9%), (iii) 10-14 cig/day (1.8%), (iv) 15-9 cig/day (1.8%) and (v) ≥ 20 cig/day (67.3%). Betel nut chewing was reported by 6 (9.0%) patients. Alcohol drinking status was categorised into non-drinkers (29.9%), ex-drinkers (1.5%), and 3 categories of chronic drinkers (i) < 10 units/week (4.3%), (ii) 11-20 units/week (19.1%) and (iii) > 20 units/week (74.5%). Advice on smoking and drinking and betel nut chewing reduction/cessation was given to all chronic users prior to surgery and their smoking and drinking status was followed-up to assess outcome.

Prior to admission, patients were fully staged. Current staging protocols in our unit requires each patient to undergo magnetic resonance imaging (MRI) of the head and neck, ultrasonographic (US) assessment of the neck nodes, and computed tomography (CT) of the chest and upper abdomen. Clinical staging showed that 13 patients had T1N0 disease, while 10 patients had T2N0 disease; Stage IV tumour was reported in 44 patients (65.7%). Tumour grading was also carried out and reported prior to treatment. Pathological analysis revealed that 13 patients had well differentiated SCC, 4 patients with well-moderately differentiated SCC, 40 patients had moderately differentiated disease, 5 had moderate-poorly differentiated carcinoma and another 5 patients had poorly differentiated disease. Nearly 60% of the cohort received postoperative radiotherapy. Each of the patients was followed-up for a minimum of 5 years.

### Statistical analysis

The outcomes of the categorical clinico-pathological variables were summarized as frequencies and percentages for the whole group of patients and the recurrence group. The numerical variables: 'age at 1^st ^diagnosis of SCC', 'depth of invasion (mm)', 'days in ICU post-treatment' and 'days in hospital post-treatment' were summarized by the median, minimal, and maximal values. Fisher's exact test were used to test for statistical significance of the findings, especially the effect of smoking and drinking reduction/cessation on survival at 3 and 5 years.

## Results

Tumour clearance was definitively achieved in 39 (58.2%) patients; unfortunately, tumour recurred in 26 patients and was treated by further resection and/or radiotherapy. The status of the surgical margins showed non-cohesive invasion in 34 (50.7%) patients, dysplasia at margin in 19 (28.4%) patients, and presence of severe dysplasia in 33 (49.3%) patients with mean depth of tumour invasion of 10.0 ± 6.5 mm (Min. 1.2, Max. 35.0). Vascular invasion was evident in 3 (4.5%) patients, while nerve invasion was identified only in a patient (Table 1).

The median days of stay in ICU was 1 day while the median days of stay in hospital was 12 days. Follow-up resulted in a 3-year survival of 46.8% and a 5-year survival of 40.4%. The causes of death were either tumour related (i.e. loco-regional or distant metastasis) or non-tumour related (e.g. pneumonia or any other cause that led ultimately to cardiorespiratory failure).

Advice on tobacco smoking reduction and cessation showed that 12 chronic smokers reduced their smoking habits to less than 5 cig/day and 13 chronic smokers stopped smoking immediately after diagnosis. All six patients with betel nut chewing habits stopped after being diagnosed with the disease. Advice on alcohol drinking reduction and cessation showed that 15 chronic drinkers reduced their alcohol intake to less than 10 units/week and 9 patients stopped completely after being diagnosed.

The causes of death of patients according to the smoking, betel nut chewing and drinking status are detailed in Table 2. The reduction of smoking and/or smoking cessation lead to significant reduction in mortality at 3 (P < 0.001) and 5 (P < 0.001) years. Also, the reduction in drinking alcohol and/or drinking cessation lead to significant reduction of mortality at 3 (P < 0.001) and 5 (P < 0.001) years.

**Table 2:** Tobacco smoking, betel nut chewing and alcohol drinking vs. mortality at 3 and 5 years.

Category	3-years survival	5-years survival	Cause of death
Non-smoker	9/12	8/12	Regional met
Ex-smoker	4/6	3/6	Non-cancer related
Chronic Smoker (< 5 cig/day)	3/3	2/3	Regional met
Chronic Smoker (5-9 cig/day)	2/6	2/6	Regional met
Chronic Smoker (10-14 cig/day)	0/1	0/1	Regional met
Chronic Smoker (15-19 cig/day)	0/1	0/1	Regional met
Chronic Smoker (≥ 20 cig/day)	4/37 (P < 0.001)	4/37 (P < 0.001)	Regional and distant met
Reduction of smoking	10/12 (P < 0.001)	9/12 (P < 0.001)	Regional and distant met
Cessation of smoking	11/13 (P < 0.001)	10/13 (P < 0.001)	Regional and distant met
Betel chewing	2/6	1/6	Regional and distant met
Non-drinker	9/20	9/20	Cancer and non-cancer
Ex-drinker	1/1	1/1	Cancer and non-cancer
Chronic drinker (< 10 u/week)	2/2	2/2	Cancer and non-cancer
Chronic drinker (10-20 u/week)	3/9	4/9	Non-cancer related
Chronic drinker (> 20 u/week)	7/35	3/35	Regional and distant met
Alcohol reduction	10/15 (P < 0.001)	9/15 (P < 0.001)	Non-cancer related
Alcohol cessation	8/9 (P < 0.001)	7/9 (P < 0.001)	Regional met

Also the effect of smoking and drinking reduction/cessation on the mortality. Causes of death have been identified

## Discussion and conclusion

At least three quarters of oral cancers could be prevented by the elimination of tobacco smoking and reduction in alcohol consumption. The removal of these two risk factors also reduces the risk of recurrence or second primary in people with oral cancer. Smoking cessation is associated with a rapid decline in the risk of oral cancers, with a 50% reduction in risk within 3 to 5 years [24].

One of the most effective ways of helping patients prevent or reduce the morbidity and mortality effects of oral cancer is by promoting tobacco and alcohol abandonment. A need for further promotion of smoking cessation activities by the health professionals was identified [25]. In particular, smoking cessation programs are needed to prevent the increase in mortality from these cancers in many low- and middle-income countries [26].

Dental and medical practitioners have a unique opportunity to lead the advancement in this field aiming to reduce the global effect of oral and oro-pharyngeal and laryngeal cancers.

Reduction or cessation of smoking soon after diagnosis reduced the rate of death significantly (P < 0.001). The same principle applies for cessation of alcohol drinking. Chewing betel can be associated with very poor prognosis and death from loco-regional or distant metastasis most likely due to a wide spread field effect.

## Competing interests

The authors declare that they have no competing interests.

## Authors' contributions

All authors have contributed intellectually and to the writing of this manuscript. AP: contributed to the primary analysis of this study and the results are highlighted in Table 1 of this study. All authors read and approved the final manuscript.
